# Comment on “Effectiveness and safety of oral vancomycin for the treatment of inflammatory bowel disease associated with primary sclerosing cholangitis: a systematic review and pooled analysis”

**DOI:** 10.1177/17562848251332453

**Published:** 2025-04-21

**Authors:** Richard Kellermayer, Peter Lewindon, Cynthia Buness, James Tabibian, Kevin Johnson, Shamita Shah, Parambir Dulai, Ahmad H. Ali, Ayesha Shah, Gerald Holtmann, Harland S. Winter

**Affiliations:** Division of Pediatric Gastroenterology, Department of Pediatrics, Texas Children’s Hospital, Baylor College of Medicine, 6621 Fannin Street, Houston, TX 77030-3411, USA; USDA/ARS Children’s Nutrition Research Center, Houston, TX, USA; University of Queensland, Brisbane, QLD, Australia; Queensland Children’s Hospital, Brisbane, QLD, Australia; Global Liver Institute Pediatric and Rare Liver Diseases Research Council, Washington, DC, USA; Autoimmune Liver Disease Network for Kids, Stanford University, Stanford, CA, USA; National Patient Advocate Foundation, Washington, DC, USA; Advanced Gastroenterology Center Adventist Health Glendale Medical Center, Glendale, CA, USA; Department of Radiology and Biomedical Imaging, Yale University, New Haven, CT, USA; Department of Gastroenterology, Ochsner Medical Center, New Orleans, LA, USA; Division of Gastroenterology and Hepatology, Northwestern University Feinberg School of Medicine, Chicago, IL, USA; Division of Gastroenterology and Hepatology, University of Missouri School of Medicine, Columbia, MO, USA; Faculty of Medicine, The University of Queensland, Brisbane, QLD, Australia; Department of Gastroenterology and Hepatology, Princess Alexandra Hospital, Brisbane, QLD, Australia; Faculty of Medicine, The University of Queensland, Brisbane, QLD, Australia; Mass General Hospital for Children, Harvard Medical School, Boston, MA, USA

**Keywords:** inflammatory bowel disease, primary sclerosing cholangitis, ulcerative colitis, vancomycin, vancomycin resistant enterococcus, VRE

To the Editor,

We read with great interest and no small concern the recent review by Sannaa et al.^1^ on the use of oral vancomycin therapy (OVT) in primary sclerosing cholangitis (PSC) and associated inflammatory bowel disease (PSC-IBD). While we are encouraged by the growing body of literature demonstrating the effectiveness and safety of OVT, we are deeply disturbed by the inaccurate report of an 8% risk of vancomycin-resistant enterococcus (VRE) carriage in patients.

We acknowledge that the authors have submitted a corrigendum to their manuscript. However, we must stress that the fear surrounding VRE emergence is such that any figure suggesting an 8% VRE risk in PSC patients treated with OVT could severely compromise—if not entirely terminate—the potential for further investment in this valuable drug within this patient population.

It is therefore crucial that this misconception is clearly and promptly corrected, and that the correction is widely disseminated before it results in our infectious disease colleagues withdrawing their support for OVT. OVT holds promise as a therapy in PSC and PSC-UC, where no other treatments have demonstrated similar potential to improve patient outcomes.^2^

We wish to restate that the finding of VRE cases in the recent review by Sannaa et al.^1^ was based on preliminary data from an abstract of a study involving only 15 patients, where VRE was prematurely ascribed to 6 individuals. The subsequently completed analysis of the study, published in 2024,^3^ confirmed that no evidence of VRE was found in these patients.

It is therefore vital to emphasize that the five studies referenced by Sannaa et al.,^1^ along with the large body of published (Table 1) and yet unpublished experience, have failed to demonstrate a single case of VRE emergence in the treatment of PSC and PSC-IBD. While the number of reported OVT treatments with VRE testing remains relatively small, the data available support that the emergence of VRE is a minimal risk both for patients and the broader community. The risk of VRE in this setting should not be overstated or incorrectly portrayed, as it could unnecessarily undermine a promising therapy for PSC and PSC-IBD.

**Table 1.** Characteristics of studies reporting clinical response/remission to oral vancomycin in PSC and PSC-IBD.

Study^a^	Study design	IBD-PSC patients (*n*)	Dose of oral vancomycin, NR	Mean/median vancomycin duration (months)	Liver/gut improvement, NR	VRE reported
Abarbanel et al., 2013	Open-label trial	14	Stanford protocol^b^	12–24 (range)	Yes for both (most)	No
Ahmed et al., 2023	Case report	1	125 mg QID	3	Yes for colon NR for liver	No
Alenchery et al., 2023	Retrospective case series	17	500–1500 mg/day; 1 patient at 3000 mg/day	6–96 (range)	NR for colon Yes for the liver (many)	No
Ali et al., 2020	Prospective open-label trial	59	Stanford protocol^b^	2.4–168 (range); 30 (median)	Yes for both (most)	No
Almomen et al., 2023	Case report	1	500 mg BID	24	Yes for colon NR for liver	No
Balouch et al., 2023	Retrospective case series	29	Stanford protocol^b^	24 (median)	Yes for the colon (most) NR for liver	No
Britto et al., 2021	Case report	1	125 mg QID	6.5	Yes for both	No
Brost et al., 2014	Case report	1	NR	0.3	Yes for both	No
Buness et al., 2021	Case report	1	Stanford protocol^b^ to 2250–2000 mg/day	384	Yes for both	No
Cox et al., 1998	Case series	3	(1) 250 mg QID (2) 125 mg TID (3) 250 mg TID	6–14 (range)	Yes for both	No
Dao et al., 2019	Retrospective case series	8	125 mg QID	9–36 (range); 8 (mean)	Yes for colon NR for liver	No
Davies et al., 2008	Case series	14	Stanford protocol^b^	54 ± 43 (mean); 30–118 (range)	10 complete, 4 did not respond	No
Davies et al., 2013	Case report	1	Stanford protocol^b^	36	NR for colon Yes for liver	No
de Chambrun et al., 2018	Retrospective case series	3	500 mg BID	12–36 (range)	2 complete; 1 colon only	No
Deneau et al., 2020^c^	Matched retrospective analysis	88	21 mg/kg/day (median); 15–29 mg/kg (range)	~5–12 (range)	Yes for the colon (NR in detail) No for liver	No
DiGiorgio, 2021^c^	Case series	12	Stanford protocol^b^	1–99 (range); 53 (median)	6 complete, 4 partial, 4 did not respond	No
Fahad et al., 2018	Retrospective case series	6	125 mg QID (4–8 weeks) then 125 mg TID	6–24	Yes for colon Yes for liver (*n* = 4)	No
Fiske et al., 2024	Case report	1	NR	13	Yes for both	No
Gomez et al., 2022	Case report	1	500 mg BID	24	Yes for colon NR for liver	No
Hey et al., 2017	Case report	1	250 mg BID	22	NR for colon Yes for liver	No
Kaya et al., 2023	Case series	4	250–500 mg BID	6–8 (range)	Yes for colon NR for liver	No
Lev-Tzion et al., 2017^d^	Case series	5	NR	0.75–15 (range)	Yes for colon (4 of 5 responded) NR for liver	No
Nguyen et al., 2024	Case report	1	500 mg BID	18	Yes for colon NR for liver	No
Perez Martin et al., 2023	Case report	1	Stanford protocol^b^	10	Yes for colon NR for liver	No
Quraishi et al., 2025^3^	Prospective open-label trial	15	125 mg QID	1	Yes for both (12 of 15 responded)	No
Rahimpour et al., 2016^c^	Randomized, placebo-controlled, triple blinded	18	125 mg QID	3	Yes for both (some)	No
Räisänen et al., 2025^d^	Observational retrospective study	44	Stanford protocol^b^	7.6	Yes for the colon (most) NR for liver	No
Rahman et al., 2021	Case report	1	125 mg BID	40	Yes for both	No
Ricciuto et al., 2021	Multicenter retrospective cohort	70	16 mg/kg/day (median dose)	30 (median); 18–49 (range)	Yes for the colon (most) NR for liver	No
Shah et al., 2022	Retrospective case series	7	250–1500 mg/day	32.1 (mean); 9–31 (range)	Yes for both (most)	No
Shen et al., 2021	Case report	1	250 mg QID	52	Yes for both	No
Sohal et al., 2024	Case report	1	Stanford protocol^b^	17	NR for colon Yes for liver	No
Tabibian et al., 2013^c^	Double-blinded, randomized trial	17	125 or 250 mg QID	3	NR for colon Yes for the liver (some)	No
Tan et al., 2019	Retrospective case series	17	NR	8.1 (mean)	Yes for the colon (most) Yes for the liver (most)	No
Tsay et al., 2019	Retrospective case series	13	NR	21.6 (mean)	Yes for both (most)	No
Total		477				

Here are 15 additional studies (not counting the 6 abstracts^a^) combined with those in Sannaa et al.^1^ None reported VRE or any adverse events.

a Six abstracts not included (*N* = 71): Beedie 2024, Cintosun 2025, El-Youseff 2017, Higley 2020, Pratt 2011, Talamantes 2024.

b “Stanford protocol”: 50 mg/kg if pt <30 kg divided into three doses; 1500 mg/day divided into three doses if pt ⩾30 kg.

c The number of patients is only those who were treated with oral vancomycin (Deneau, DiGiorgio, Rahimpour, and Tabibian).

d Atypical UC is defined as reverse gradient, right-sided, or patchy colitis with/without rectal sparing and/or backwash ileitis, and expressing atypical ANCA serology (pANCA).

IBD, inflammatory bowel disease; NR, not reported; PSC, primary sclerosing cholangitis; VRE, vancomycin-resistant enterococcus; BID, twice daily; QID, four times daily; TID, three times daily.

## Declarations

**Ethics approval and consent to participate:** Not applicable.

**Consent for publication:** Not applicable.

**Availability of data and materials:** Not applicable.

**Declaration of generative AI and AI-assisted technologies in the writing process:** During the preparation of this work, the author(s) used ChatGPT to improve language and readability. After using this tool/service, the author(s) reviewed and edited the content as needed and take(s) full responsibility for the content of the publication.
